# Molecular Mechanisms of Enzalutamide Resistance in Prostate Cancer

**DOI:** 10.1007/s40610-017-0079-1

**Published:** 2017-10-23

**Authors:** Zoran Culig

**Affiliations:** Experimental Urology, Department of Urology, Medical University of Innsbruck, Anichstrasse 35, A-6020 Innsbruck, Austria

**Keywords:** Anti-androgens, Enzalutamide, Truncated androgen receptor, Mutations, Cytokines

## Abstract

**Purpose of Review:**

Compensatory mechanisms leading to increased androgen receptor expression and activity after androgen ablation or anti-androgen treatment have been identified in prostate cancer. After hydroxyflutamide and bicalutamide were used in therapy of prostate cancer over many years, novel anti-androgen enzalutamide showed improved clinical activity. However, enzalutamide resistance develops over a certain time period, and molecular mechanisms responsible for this process are heterogeneous.

**Research Findings:**

As with other anti-androgens, these mechanisms include alterations of AR but also may be associated with overexpression of oncogenes which should be targeted by novel therapies. Androgen receptor splice variants have been frequently described in patients who developed enzalutamide resistance. Mutant AR F876L has been detected in patients who are resistant to enzalutamide. Glucocorticoid receptor overexpression has been observed in patient tissues and in pre-clinical models of enzalutamide resistance.

**Summary:**

There is a heterogeneous picture of enzalutamide resistance in prostate cancer and, therefore, the development of appropriate post-enzalutamide treatment remains a challenge.

## Introduction

The role of androgen receptor (AR) in castration therapy-resistant prostate cancer is a subject of intensive investigation in urological oncology. It is well-known that the expression of AR may critically influence cancer progression. Androgen ablation therapy and blockade of AR are considered palliative treatments for non-organ confined prostate cancer. Anti-androgens hydroxyflutamide and bicalutamide have been used over many years in prostate cancer treatment, and the studies on resistance mechanisms were particularly focused on AR mutations which emerge during endocrine therapy with these compounds. Alterations in expression and function of several AR coactivators are also relevant for acquisition of resistance to anti-androgens in prostate cancer. The development of novel anti-androgenic compounds resulted in a worldwide use of the non-steroidal anti-androgen enzalutamide, a drug that binds to the ligand-binding domain of the AR thus preventing nuclear transactivation and expression of target genes. Several clinical studies with enzalutamide revealed that the use of this anti-androgen leads to improved survival of prostate cancer patients [1]. However, resistance towards enzalutamide occurs and models have been developed in order to investigate the underlying mechanisms. This review will therefore focus on models and mechanisms relevant to resistance to enzalutamide in human prostate cancer. Since alternative strategies on the basis of pre-clinical models for targeting therapy resistance have been proposed, they will also be presented in this review.

## Androgen Receptor Splice Variants

Constitutively active ARs are of special interest in castration therapy-resistant prostate cancer. These receptors lack ligand-binding domain and exhibit transcriptional activity in the absence of androgenic hormones. Li and associates [2•] demonstrated that inhibition of growth of CWR-R1 and 22Rv1 cells could not be achieved by bicalutamide or enzalutamide. The cells are known for enriched expression of truncated AR. These anti-androgens inhibited transcriptional activity of full-length AR in 22Rv1 cells. However, inhibition of full-length AR has no effect on growth of these cell lines. Growth retardation was re-established following knockdown of the variant AR. The authors have demonstrated that the genes responsive to AR activation were constitutively regulated by truncated ARs [2•]. Variant ARs regulate expression of a subset of AR target genes which are distinct to those regulated by full-length AR. Genes affected are relevant to the M phase of cell cycle. The importance of constitutively active ARs in acquisition of enzalutamide resistance could be confirmed in studies with EPI-002 which targets the N-terminal region of the AR [3]. That domain of the AR is a location for transcription activation function-1 of the receptor. The effects of EPI-002 could be in part explained by inhibition of interaction between the receptor and the coactivator CBP. This fact is important because of increased expression of CBP during androgen ablation therapy [4]. In connection with regulation of enzalutamide sensitivity and resistance, EPI-002 inhibits transcriptional program regulated by truncated AR and diminishes the expression of V7. Consistent with in vitro findings, in vivo growth of the enzalutamide-resistant xenograft LNCaP 95 was inhibited by EPI-002. Immunohistochemical analyses confirmed decreased proliferation and enhanced apoptosis in those xenografts treated with EPI-002. It is worthwhile to mention that a prodrug of EPI-002, EPI-506, has been used in clinical studies phase I/II. Additional effect could be achieved by targeting the mTOR pathway in enzalutamide-resistant prostate cancer as evidenced by the use of BEZ235, a dual inhibitor of phosphatydilinositol 3-kinase and TORC 1/2 [5]. This cotargeting approach in PTEN-negative enzalutamide-resistant cells was not associated with wide prohibitive side effects.

Inhibition of variant AR V7 could be achieved with niclosamide [6•]. Niclosamide has been selected by screening Prestwick Chemical Library, which contains about 1120 small molecules. Luciferase assays were used for determination of receptor activity. Niclosamide is an antihelminthic drug which significantly inhibited expression of V7, its transcriptional activity, recruitment of receptor to the promoter of the prostate-specific antigen gene, and in vivo growth of enzalutamide-resistant C4-2 tumors. AR variant inhibition could be also achieved by cotreatment with niclosamide and the androgen synthesis inhibitor abiraterone [7]. Inhibition of expression of V7 is achieved by enhanced degradation of the protein. Clinical evaluation of AR-V7 in therapy resistance was also performed [8]. Patients with considerable expression of AR-V7 in circulating tumor cells were found to have a lower response rate compared to AR-V7-negative patients. So far, the pre-clinical and clinical evidence that AR-V7 is implicated in resistance to enzalutamide is conclusive. Patients with a higher nuclear expression of AR-V7 protein in circulating tumor cells may be good candidates for treatment with taxanes, based on overall survival data [9].

## Androgen Receptor Mutations

In case of hydroxyflutamide and bicalutamide treatment, several mutations have been discovered during chronic treatment with the drugs [10, 11]. In some cases, functional characterization of mutated receptors has revealed their involvement in resistance. In experiments in which mutated AR cDNA was expressed in cancer or other cell lines, it could be demonstrated that anti-androgens caused a higher transactivation in the presence of mutated AR. It was shown that the missense AR F876L mutant, detected in plasma DNA, is sufficient for cells to acquire resistance to enzalutamide and the anti-androgen ARN-509 [12••]. Both enzalutamide and ARN-509 exhibited agonistic activity in the presence of mutated AR and promoted interaction between the N- and C-terminal domains of the receptor. In vivo growth of tumors bearing mutated AR could not be inhibited by enzalutamide. The findings open the possibility to target that specific mutation in order to overcome enzalutamide resistance. Similar conclusions about the importance of that mutation for acquisition of enzalutamide resistance were reached by Korpal and colleagues [13••]. On the basis of results of those studies, screening of the patients’ tissues to predict sensitivity to enzalutamide may be expected. Cells expressing the mutated AR were however sensitive to cyclin-dependent kinase inhibitors LEE011 and PD033299, most probably on the basis of their inhibition of dysregulated E2F1 function.

## Glucocorticoid Receptor

Importantly, glucocorticoid receptor expression is increased in cells which become resistant to enzalutamide therapy [14]. Adaptive resistance to enzalutamide by enhanced expression and function of glucocorticoid receptor is recognized on the basis of pre-clinical and clinical studies. This issue is particularly interesting because of use of glucocorticoid drugs in therapy of prostate cancer. Glucocorticoids are used to suppress the levels of pituitary adrenocorticotropic hormone and decrease production of adrenal androgens. Glucocorticoid receptor agonists may thus contribute to therapy resistance, and the potential of receptor antagonists has to be further examined. However, it should be kept in mind that glucocorticoid receptor antagonists, such as RU 486, may cause partial agonism of the AR. In resistant tumor tissues, glucocorticoid receptor could increase the expression of AR target genes thus contributing to cancer progression, consistent with overlapping transcriptomes and cistromes for both receptors. Glucocorticoid receptor is also implicated in resistance to chemotherapy in prostate cancer [15]. Glucocorticoid receptor antagonists could revert resistance to docetaxel in several prostate cancer cellular models. In conclusion, targeting glucocorticoid receptor in prostate cancer may be a strategy that will affect growth of a larger subgroup of tumors. In view of the negative effect of docetaxel on AR activity, one should consider the issues of resistance to chemotherapy in models that do not respond to enzalutamide [16, 17]. For this purpose, enzalutamide-resistant cell lines were subject to treatment with microtubule inhibitors [18]. The researchers showed that the efficacy of chemotherapeutics is impaired in conditions of enzalutamide resistance. Thus, enzalutamide resistance is a considerable therapeutic challenge, and future studies will likely examine individualized use of combination of agents that have a potential to target enzalutamide-resistant cancer cells on the basis of predictive markers. If cabazitaxel, a drug that is developed after docetaxel, is applied in therapy for metastatic prostate cancer, the effect on enzalutamide-resistant cells may be preserved [19]. The results could be explained by a higher potency of cabazitaxel in suppression of microtubule dynamics.

There are several other interactions between molecules involved in regulation of chemosensitivity and androgen signaling pathway in enzalutamide resistance. Twist is a transcription factor that is implicated in regulation of epithelial to mesenchymal transition and docetaxel resistance. Twist itself is up-regulated during androgen ablation therapy. Inhibition of Twist signaling was found to be associated with enhanced efficacy of enzalutamide therapy [20]. This could be achieved by use of the protein kinase C inhibitor Ro31-8220 that subsequently down-regulates Twist which has been tested in parental LNCaP cells and their derivative C4-2 as well as in 22 Rv1 cells. Enhanced phosphorylation of protein kinase C has been observed in enzalutamide-resistant tumors. The question whether this approach is effective in clinical settings remains to be answered. Resistance to enzalutamide is also promoted by Snail, which is highly expressed in aggressive prostate cancer, through regulation of AR activity [21]. Snail expression is also increased in tumor tissues obtained from patients who do not respond to enzalutamide. Interestingly, Snail up-regulates the expression of wild-type and constitutively active AR. Consequently, Snail is also responsible for enhanced migration and invasion properties in prostate cells that became resistant to enzalutamide. On the other hand, anti-androgens may be efficient in regulation of resistance to chemotherapy. The effectiveness of docetaxel in target cells is dependent on the presence of ABCB1 which belongs to the ATP-binding cassette transporters and transport substrates, such as taxanes to regulate efflux of the drugs. Activity of ABCB1 is down-regulated by bicalutamide and enzalutamide [22] thus leading to resensitization to docetaxel. Interestingly, the effect of bicalutamide on resensitization could be observed both in AR-positive and -negative cells. These results may provide scientific basis for use of anti-androgens in therapy of patients who failed chemotherapy.

## Cytokines and Enzalutamide Resistance

Expression of several cytokines in normal and malignant tissue is under control of nuclear factor kappa B. It is particularly important that interleukins (IL) are involved in regulation of many functions in prostate cancer, and IL-6 is highly expressed in castration-resistant tumors. IL-6 increases transcriptional activity of the AR in a ligand-independent and synergistic manner with low concentration of androgens. Experimental therapies against IL-6 have been established; however, clinical trials were performed in patients with late stage prostate cancer and were not successful [23, 24]. Nuclear factor kappa B is the master regulator of expression of several cytokines. The p52 subunit of nuclear factor kappa B also contributes to the development of enzalutamide resistance [25•]. In cells generated by chronic treatment with enzalutamide, down-regulation of the p52 subunit is associated with resensitization to enzalutamide. The p52 subunit of nuclear factor kappa B is also implicated in up-regulation of splice variants of the AR and glucose metabolism [26]. The effect of p52 on splice variants of the AR may be mediated by heterogenous nuclear RNA-binding protein (hnRPA-1). In this context, down-regulation of hnRPA-1 may lead to resensitization of enzalutamide-resistant prostate cancers. Cells that overexpress p52 increase glucose uptake and produce higher ATP and lactate levels. IL-6 acts through the Janus kinase (JAK)–signal transducer and activator of transcription (STAT) pathway and anti-STAT3 approaches have been proposed in several human cancers, including prostate cancer. Treatment of prostate cancer cells with the JAK inhibitor AG490 lead to resensitization of cells to enzalutamide [27]. Several aspects of JAK/STAT inhibition may be considered. Importantly, timing of this treatment may be critical. One could hypothesize that early application of potential drugs targeting the JAK/STAT pathway could delay the development of enzalutamide resistance. Liu and colleagues have demonstrated that cotreatment with enzalutamide and AG490 has an inhibitory effect on cell growth and induces apoptosis [27]. Since STAT3 is implicated in regulation of multiple cellular functions, its inhibition may also lead to down-regulation of processes leading to epithelial to mesenchymal transition in prostate cancer. Signaling of cytokines is normally under control of suppressors of cytokine signaling and protein inhibitors of activated STAT (PIAS). These molecules may have multiple functions and also participate in other signaling pathways. Thus, it was demonstrated that PIAS1 has an important role in prevention of degradation of the AR in vitro [28]. When PIAS1 was down-regulated, the effect of enzalutamide on inhibition of prostate cancer cells was enhanced. Previous studies revealed that PIAS1 is regulated by AR, and its expression is increased in prostate cancer [29]. Modulation of response to enzalutamide could also be achieved through by suppressor of cytokine signaling (SOCS)-3 [30]. Chronic treatment with enzalutamide resulted in enhanced expression of stemness-associated genes such as SOX2 and NANOG. Modulation of SOCS-3 expression, which is involved in regulation of activities of IL-6, is sufficient to counteract the effect of enzalutamide on stemness genes in cancer cells. Taken together, those results show that the network of cytokines, their receptors, and endogenous inhibitors of signaling has an impact on anti-androgen resistance in prostate cancer.

## Potential Post-enzalutamide Therapies

There may be different approaches on how to develop post-enzalutamide therapies. An important issue that should be critically discussed is administration of novel anti-androgens. Although important, the duration of post-enzalutamide response in such a case will be rather limited. The AR itself may be a primary target of therapy of enzalutamide-resistant cancers. The use of novel AR antisense oligonucleotides targeting exon 1 and exon 8 has revealed that there is a therapeutic potential to target resistant tumors [31]. This experimental therapy was found to be effective in an LNCaP subline as well as in a patient-derived xenograft. The effect of oligonucleotides may be potentiated by cotargeting clusterin in anti-androgen-resistant models [32]. Anti-clusterin therapy was proposed on the basis of cytoprotective effects of that chaperone that is regulated by the AR. Several models of enzalutamide-resistant cells have been generated and alterations in expression of AR have been documented [33]. Amplification of the AR gene in conditions of enzalutamide resistance has been observed in the LAPC-4 model whereas increased expression of the AR protein in the resistant DUCaP subline may be based on activation of other regulatory mechanisms. Further evidence has been obtained to show that both AR and non-AR pathways have been implicated in acquisition of therapy resistance in prostate cancer in models that exhibited increased metastatic growth [34].

Thus, compound 30, developed by optimizing receptor-binding efficiency of aryloxy tertramethylcyclobutyl lead compounds identified in a high throughput cellular screen, was found to suppress growth of castration (LNCaP C4-2)- and enzalutamide-resistant cells in vitro and in vivo [35]. The mechanism of inhibition of growth includes down-regulation of AR transcriptional activity. Further studies may be conducted in order to determine appropriate time schedule of drug use and interactions with other anti-prostate cancer compounds. Enhancement of enzalutamide-activity may be achieved by treatment with other drugs, such as methylselenolol prodrug [36]. Cotreatment with methylselenolol and enzalutamide suppressed AR signaling more efficiently than each drug alone. Whether methyleselenolol could negatively affect growth of established enzalutamide-resistant cells remains to be investigated. As mentioned above, an important aspect of improvement of clinical treatment beyond AR inhibition is a design of novel compounds which antagonized mutated receptor F876L substitution [37•]. In this context, Korpal and colleagues showed that cyclin-dependent kinase 4/6 inhibitors antagonize AR F876L function [13••].

Another approach to reverse enzalutamide responsiveness is targeting autophagy. Nguyen and associates showed that treatment of cells with enzalutamide induced autophagy in prostate cellular models [38]. Autophagic process is mediated by AMP-dependent protein kinase and suppression of mammalian target of rapamycin. In cells in which AMP-dependent protein kinase and, subsequently autophagy, are inhibited, therapy with enzalutamide may become more efficient. Modulation of autophagy has been considered a molecular target in cancer therapy. This approach has been proposed on the basis of in vitro and in vivo data. A significant inhibition of tumor size was measured in cells treated with enzalutamide and the inhibitor of autophagy CMI, a drug that is approved in clinical medicine for treatment of depression.

A common feature of resistance to several types of androgen deprivation is up-regulation of activity of Akt. Enzalutamide treatment may be therefore enhanced if an inhibitor of the phosphotidylinositol 3 kinase-Akt pathway AZD5363 is combined with enzalutamide [39]. This cotreatment inhibits cell cycle progression and induces apoptosis in LNCaP cells and their C4-2 derivative. In consequence, cotreatment with AZD5363 delays the development of resistance to enzalutamide. Enzalutamide therapy was also combined with the MET kinase antagonist cabozantinib [40]. MET kinase is suppressed by androgen signaling and highly regulated by androgen ablation. That study may provide a basis for improvement of clinical trials with cabozantinib in castration-resistant prostate cancer.

Further studies may be focused on the role of a potential target in immunotherapy, PD-L1 in enzalutamide resistance. PD-L1 is expressed at a high level in enzalutamide-resistant prostate tumors [41, 42]. Xenografted tumors that express high levels of PD-L1 have also been generated. One of potentially negative aspects of enzalutamide treatment may include up-regulation of HER-2 after continuous treatment. Experimental treatments of castration therapy-resistant cells with the anti HER-2 drug lapatinib yielded an anti-tumor effect.

Bromodomain (BET) inhibitors target bromodomain-containing proteins BRD/2/3/4 and BRDT. They enhance efficacy of therapy and disrupt resistance to enzalutamide in prostate cancer treatment as evidenced in the LNCaP and VCaP models [43]. Multivalent peptoid conjugates were also proposed to overcome enzalutamide resistance in prostate cancer [44]. BIRC6, a member of inhibitor of apoptosis pathways, could be also a target in enzalutamide resistance [45]. A pyrrole-imidazole polyamide is also active in these conditions [46].

## Conclusions and Future Perspective

Replacement of a single anti-androgen with another one is a task which could in most clinical cases lead to a temporary improvement of clinical symptoms and biochemical status. This approach could be in part useful in patients who failed enzalutamide treatment. As elaborated in this review, several mechanisms related to changes in AR itself have to be considered in patients with enzalutamide resistance. Most importantly, AR mutation(s) certainly contribute to drug resistance. However, identification of novel targets will likely lead to therapies which are not solely limited to AR inhibition. Similarly as with other mechanisms related to anti-androgen resistance, development of enzalutamide resistance seems to be very heterogeneous. This factor strongly limits durable success in therapy for castration therapy-resistant prostate carcinoma.
